# Hibernomas: a rare benign lipoma subtype

**DOI:** 10.1093/jscr/rjad472

**Published:** 2023-08-29

**Authors:** Runze Wei, Zhaolei Chen

**Affiliations:** Department of Vascular Surgery, Northern Jiangsu People's Hospital, Yangzhou, China; Department of Vascular Surgery, Northern Jiangsu People's Hospital, Yangzhou, China

**Keywords:** hibernoma, diagnosis, brown fat tumor

## Abstract

Hibernoma is a benign soft tissue tumor; it is extremely low in incidence, slow-growing and painless and is often mistaken for lipoma or liposarcoma. Diagnosis requires imaging and pathological analysis. Complete resection is the treatment of choice. We analyzed a case of lipoma from imaging and pathology perspectives and reviewed the relevant literature.

## INTRODUCTION

Hibernomas, also known as brown adipose tumors, are benign tumors originating from brown adipose cells. They are characterized by slow growth and painlessness, often going unnoticed. The incidence rate of hibernomas among adipose cell tumors is ~1.1% [1]. In this article, we aim to enhance understanding of this type of tumor and prevent misdiagnosis by reporting the clinical data of a case involving an inguinal artery aneurysm and reviewing relevant literature.

## CASE REPORT

**Figure 1:**
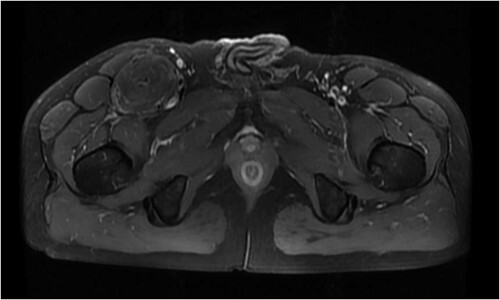
Equal and slightly high signal in T1W1 + C.

**Figure 2:**
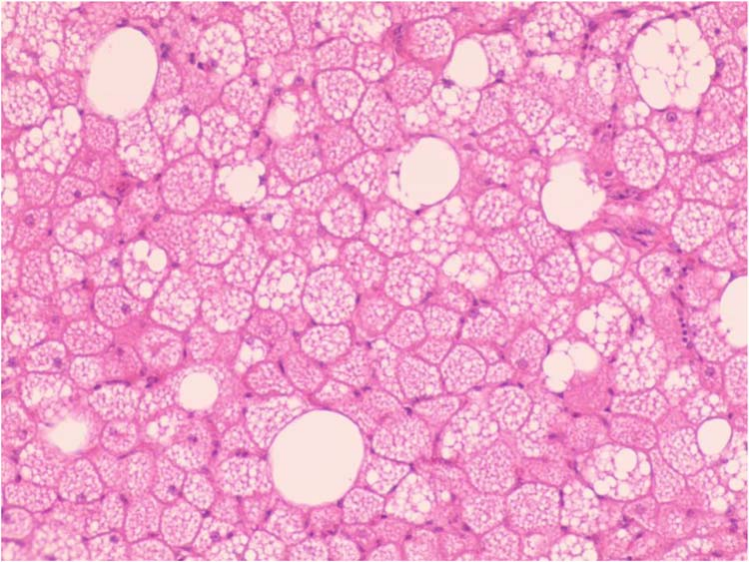
Hypertrophic adipoid cells with abundant interstitial vessels (HE^*^400).

The patient was admitted to the hospital due to the discovery of an inguinal mass that had been present for 1 year. The patient initially noticed swelling in the groin area a year ago. Over time, it gradually increased in size and caused localized discomfort. During the examination, a mass the size of a hen’s egg was felt in the right inguinal region. It had limited mobility and was not tender. No significant abnormalities were found in the motion or sensation of the right lower limb. Ultrasonography showed an isoechoic mass within the muscle tissue of the left inguinal region. Unenhanced and enhanced magnetic resonance imaging (MRI) scans suggested a well-defined intermuscular mass at the root of the right thigh. The mass showed low signal intensity on diffusion-weighted imaging and isointense-to-slightly hyperintense signal post-enhancement (Fig. 1). The patient then underwent excisional biopsy. The excised tissue showed a capsulated, lobulated, soft and greasy mass with a brownish-yellow cut surface. Ultrastructural examination revealed large multivacuolated adipocytes with eccentric nuclei, cytoplasmic acidophilic granules and small lipid droplets. Additionally, abundant blood vessels were present within the stroma. Immunohistochemistry results showed positive staining for S100 protein and negative staining for CD34 (Fig. 2).

## DISSCUSSION

Hibernomas are rare adipocytic tumors that primarily affect individuals aged 30–40 years old. They are commonly found in regions rich in brown adipose tissue, such as axillary fossae, necks and interscapular areas. Cases have also been reported in abdominal regions, thighs and buttocks. Hibernomas rarely undergo malignant transformation, with only a minority of cases recurring due to incomplete excision [2]. Clinically, hibernomas present as slow-growing, painless masses. As they grow, they may exert pressure on surrounding tissues, leading to symptoms of vascular and neural compression. Commonly used imaging modalities include ultrasonography, computed tomography (CT), MRI and positron emission tomography combined with CT (PET/CT), with angiographic examination as an auxiliary tool. Ultrasonography reveals well-defined borders and high-echoic lesions. Doppler imaging allows observation of abundant blood flow signals. CT scans without contrast typically show well-circumscribed, lobulated soft tissue masses with density similar to subcutaneous fat, but with slight variation. Detailed examination may reveal linear or curvilinear internal septations. On T1- and T2-weighted MRI images, hibernomas appear isointense or slightly hyperintense. In certain regions, fat-suppressed sequences show high signal intensity. After contrast enhancement, heterogeneous enhancement patterns are commonly observed [3]. However, differentiating hibernomas from liposarcomas remains challenging using CT and MRI.

Due to the high metabolic activity and abundance of mitochondria in brown adipomas, they exhibit elevated FDG uptake on PET scans. Simultaneous CT scans show fatty masses without infiltration into surrounding tissues. PET/CT can aid in differentiating hibernomas from liposarcomas to some extent. Nevertheless, definitive diagnosis still requires histopathological confirmation. Direct observation of brown adipomas revealed irregularly lobulated, well-demarcated and pliable oily masses with diameters typically ranging from 5 to 10 cm. The cut surfaces appeared yellow-to-reddish-brown with occasional hemorrhagic areas. Under the microscope, encapsulated structures consisting of multivacuolated and univacuolated cells were observed. Immunophenotypically, brown adipoma cells were positive for Vimentin and S-100, but do not express CD34. Molecular markers such as MDM-2, CDK-4 and p-16 may provide additional diagnostic support for brown adipomas [4].

In summary, brown adipomas are a rare benign lipoma subtype that is often misdiagnosed as conventional lipomas or liposarcomas based on clinical and radiological findings. Accurate diagnosis relies on pathological analysis.

## CONFLICT OF INTEREST STATEMENT

None declared.

## FUNDING

None.
